# Characteristics and Superspreading Potential of Andes Virus Person-to-Person Transmission

**DOI:** 10.3201/eid3210.260908

**Published:** 2026-10

**Authors:** Zihao Guo, Kailun Pan, Yu Zhao, Sheikh Taslim Ali, Kai Wang, Lirong Cao, Zhuang Cui, Shengqiang Liu, Ka Chun Chong, Daihai He, Shi Zhao, Yuantao Hao

**Affiliations:** Tianjin Medical University School of Public Health, Tianjin, China (Z. Guo, K. Pan, Z. Cui, S. Zhao); The Chinese University of Hong Kong JC School of Public Health and Primary Care, Hong Kong, China (Z. Guo, L. Cao, K.C. Chong); Ningxia Medical University School of Public Health, Yinchuan, China (Y. Zhao); The University of Hong Kong, Hong Kong (S.T. Ali); Xinjiang Medical University Department of Medical Engineering and Technology, Urumqi, China (K. Wang); Tiangong University School of Mathematical Sciences, Tianjin (S. Liu); The Hong Kong Polytechnic University Department of Applied Mathematics, Hong Kong (D. He); Peking University Center for Public Health and Epidemic Preparedness and Response, Beijing, China (Y. Hao); Peking University School of Public Health, Beijing (Y. Hao); Peking University Ministry of Education Key Laboratory of Epidemiology of Major Diseases, Beijing (Y. Hao)

**Keywords:** Andes virus, viruses, person-to-person transmission, hantavirus, hantavirus pulmonary syndrome, respiratory infections, risk assessment, effective reproduction number, disease superspreading, serial infection interval

## Abstract

By using historical contact tracing data, we estimated that 23.4% of case-patients caused 80% of Andes virus (ANDV) person-to-person transmission. We demonstrated a low but nonnegligible probability of observing a large-scale ANDV infection outbreak in a rodent-free setting consisting of close contacts, despite the historically self-limited person-to-person transmission of ANDV.

Hantaviruses are rodentborne zoonotic enveloped RNA viruses belonging to the family Hantaviridae (*1*). The genus *Orthohantavirus*, which belongs to the subfamily Mammantavirinae, is the only genus in Hantaviridae that can cause disease in humans (*1*). Infections in humans occur mainly through inhalation of aerosolized excreta from infected rodents, which can lead to hantavirus pulmonary syndrome, a severe respiratory disease with a case-fatality rate of up to 50% in the Americas, depending on the infecting hantavirus type (*2*). Although uncommon, person-to-person transmission of Andes virus (ANDV), a member of the genus *Orthohantavirus*, has been documented in South America in recent decades, typically in high-intensity close-contact settings, such as households and social gatherings (*3*–*9*).

On May 2, 2026, an ANDV outbreak aboard the MV Hondius cruise ship was reported to the World Health Organization (WHO), with the first case illness onset on April 3 (*10*). Subsequent epidemiologic investigations proposed a working hypothesis that person-to-person transmission originated from a seed case in a person who probably acquired infection through environmental exposure during travel in Argentina before boarding the ship on April 1 (*10*). By July 2, a total of 13 ANDV cases had been identified, including 12 laboratory-confirmed and 1 probable case; 3 of those cases were fatal (*10*). According to the initial WHO notification for the outbreak on May 2, public health and control measures, including contact tracing, case isolation, clinical care, and medical evacuation of symptomatic passengers, had been implemented onboard the ship (*11*). By using historical contact tracing data, we aimed to estimate key epidemiologic characteristics of person-to-person transmission of ANDV, including the serial interval (SI) distribution and transmission heterogeneity, given that those traits strongly shape outbreak size.

## The Study

We conducted a literature review of historical ANDV outbreak investigations and extracted contact tracing data from ANDV case clusters that involved person-to-person transmission events. We only included studies conducted in Argentina, given that the seed case-patient of the 2026 outbreak was most likely infected there, according to the WHO report (*10*). We identified 5 eligible studies that provided detailed contact histories and viral genetic evidence strongly supporting person-to-person transmission and linking infections to specific viral lineages or strains (*3*–*7*). We extracted symptom onset dates to calculate the SI and compiled case-cluster sizes to estimate the effective reproduction number (R_t_) and transmission heterogeneity measured by a dispersion parameter (k) (*12*). In case-clusters, we considered terminal cases and sporadic cases attributed to environmental exposure but with no documented human contact as generating no secondary infections.

We used a Bayesian hierarchical model to estimate the epidemiologic parameters while accounting for random effects attributable to epidemiologic differences among ANDV lineages or strains during different epidemic periods. We further conducted a branching process simulation study to assess the transmission potential of the current outbreak on the MV Hondius cruise ship on the basis of the pooled estimates of epidemiologic parameters, considering a plausible range of the initial R_t_ for ANDV (Appendix).

We identified a total of 88 case-clusters from the included studies, among which 50 (56.8%) were not associated with any secondary person-to-person transmission events. After accounting for variations across lineages or strains, the pooled estimate of R_t_ was 0.74 (95% credible interval [CrI] 0.28–1.29) and the estimate of k was 0.64 (95% CrI 0.36–1.16). Apart from inherent differences among ANDV lineages or strains, the substantial variation in R_t_ across lineages or strains could also be attributed to differences in study settings and individual-level heterogeneity in reproduction numbers (*5*). We estimated that 23.4% (95% CrI 15.1%–30.3%) of the cases generated 80% of the transmission events, suggesting the person-to-person spread of ANDV exhibited relatively high heterogeneity, although it appeared lower than that observed for other pathogens known for superspreading events, including SARS-CoV, SARS-CoV-2, Middle East respiratory syndrome coronavirus, and Ebola virus (*13*,*14*).

Among 38 case-clusters that involved secondary transmissions, we identified 76 primary–secondary transmission pairs with available symptom onset dates, which enabled us to calculate the SI. We estimated a pooled mean SI of 21.92 (95% CrI 18.97–25.78) days and an SD of 6.98 (95% CrI 5.80–8.66) days. Although we observed little variation in the SI distributions across lineages or strains, our estimates are conditional on reported case-clusters and might not represent the full population-level distribution of ANDV infections. That condition is particularly relevant given that only 1 transmission pair was identified for the ANDV Cent BsAs lineage (Figure 1).

**Figure 1:**
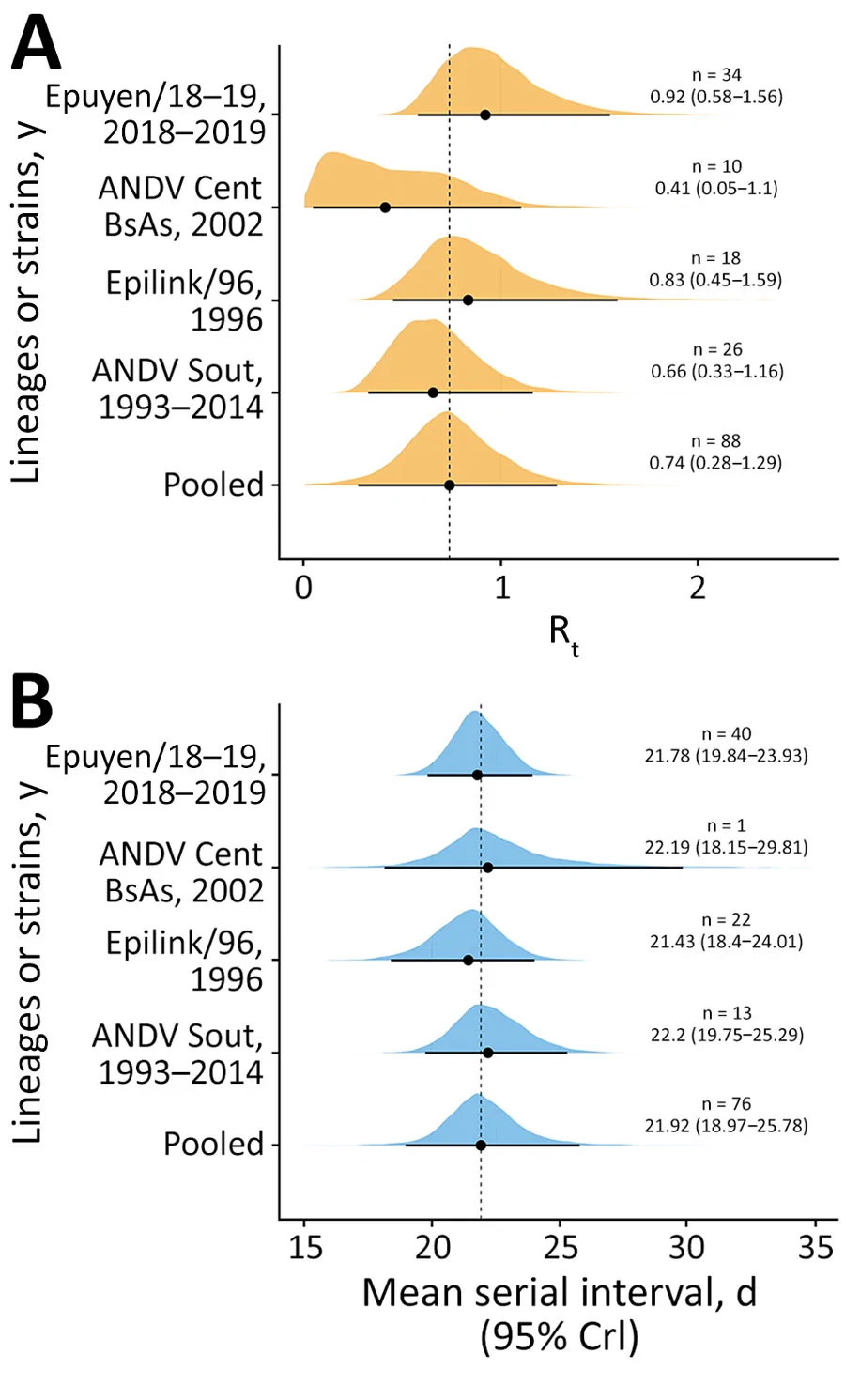
Transmission characteristics from a study of superspreading potential of ANDV person-to-person transmission. A) Pooled R_t_ estimated from historical interhuman transmission events, incorporating random effects from different ANDV lineages or strains reported in outbreaks that occurred in previous years. B) Pooled mean serial intervals across different ANDV lineages or strains. Values indicate mean (95% CrI). Dots represent mean estimates; whiskers indicate 95% CrIs. Crl, credible interval; ANDV, Andes virus; R_t_, effective reproduction number.

Assuming the initial reproduction number of the current ANDV outbreak lies within the 0.4–2.5 range (*3*) (Appendix Figure 1), together with the pooled SI and k estimates from historical data, we simulated plausible outbreak trajectories under a stochastic branching process framework. On the basis of the assumption that effective control measures were implemented after the WHO notification dated May 2, 2026 (*11*), we assumed the R_t_ decreased from that date onward (Appendix). Within 10 weeks after symptom onset of the seed case, 95% of the simulation results produced <15 cumulative cases of ANDV infection (Figure 2). We estimated the probability of sustained transmission after 1 generation of spread to be 20.9% (95% CrI 5.3%–37.4%) for historical ANDV infection clusters and 28.6% (95% CrI 10.0%–57.4%) using the assumed initial reproduction numbers of the ANDV infection outbreak on the cruise ship (Figure 3). In sensitivity analyses, excluding sporadic cases from historical clusters yielded results similar to those of the main analysis (Appendix Figure 2). The absence or delayed implementation of control measures would increase the likelihood of a large-scale outbreak, under the assumption that such measures effectively reduce the reproduction number (Appendix Figure 3).

**Figure 2:**
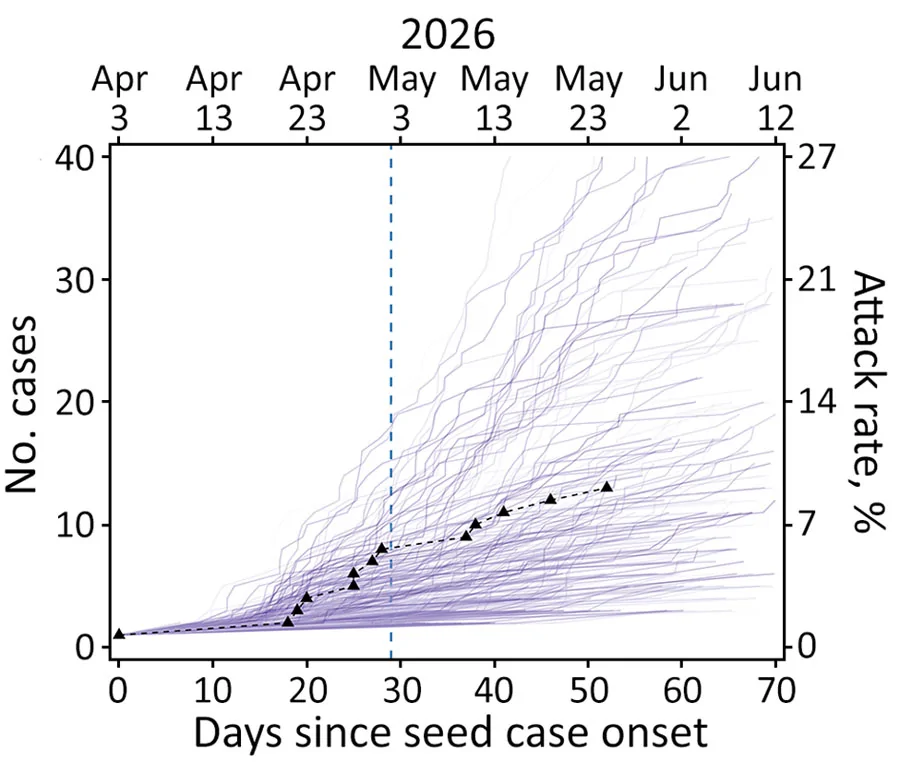
Projected outbreak trajectories from a study of characteristics and superspreading potential of Andes virus person-to-person transmission. Graph shows 1,000 branching process simulations within 10 weeks after symptom onset of the seed case (April 3, 2026), using a plausible range of the initial reproduction number (0.4–2.5) of the MV Hondius cruise ship cluster and pooled dispersion parameter (k) estimates from historical data. Symptom onset dates for secondary cases were randomly sampled from the pooled serial interval distribution. Black triangles indicate the cumulative number of cases observed in the cruise ship outbreak as of May 27, 2026. The blue dashed vertical line marks the initial World Health Organization notification date of the outbreak (May 2, 2026), after which effective control measures were assumed to have been implemented (i.e., the reproduction number was assumed to decrease). The right y-axis shows the corresponding attack rate based on a ship population of 147.

**Figure 3:**
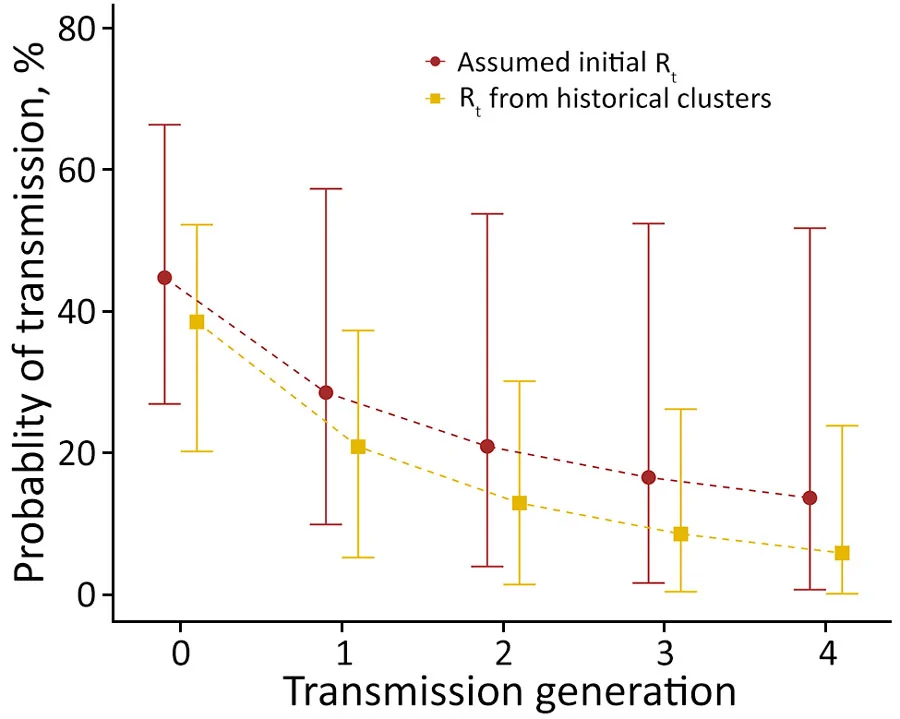
Probability of onward transmission from a study of characteristics and superspreading potential of Andes virus person-to-person transmission. Graph shows probability of onward transmission after a given number of transmission generations, estimated by using the assumed plausible initial R_t_ for the cruise ship cluster, the pooled R_t_, and the pooled dispersion parameter (k) estimates from historical clusters. Dots and squares represent mean estimates; whiskers indicate 95% credible intervals. R_t_, effective reproduction number.

Because only 8 cases occurred within approximately 1 mean SI after the putative seed case-patient’s symptom onset (18–25 days) before the initial WHO notification on May 2, 2026, conventional methods cannot reliably estimate the initial reproduction number without support of contact tracing data. Nonetheless, we used a branching process simulation model with transparent assumptions to illustrate plausible epidemic trajectories for the ANDV outbreak. We noted that person-to-person transmission of ANDV in the general community might be close to the situations of historical ANDV outbreaks, where we observed mostly self-limited risks. Timely and accurate identification of ANDV infection is critical yet challenging because of the nonspecific nature of early symptoms, which necessitates systematic risk assessment for symptomatic persons and their contacts.

A key limitation of this study was its reliance on self-reported contact histories and symptom onset dates derived from publicly available data sources. Potential misclassification of epidemiologic links attributable to unreported co-exposures to rodents, underdetection of subclinical or asymptomatic infections caused by terminal cases, and recall bias in symptom onset might have affected the parameter estimates (*15*). Of note, no clear evidence supporting transmission from asymptomatic or presymptomatic case-patients currently exists. In the simulation study, we anchored control measures to the symptom onset date of the seed case-patient, thereby overlooking the biological delay imposed by the incubation period.

## Conclusions

In summary, our results provide insights into the person-to-person transmission potential of ANDV, which exhibited substantial heterogeneity. We found a low but significant chance of observing a relatively large-scale ANDV outbreak in a rodent-free setting consisting of close contacts, which might be eradicated across a few generations of person-to-person transmission. Because no vaccine or antiviral treatment for ANDV infection is currently available, continuous surveillance of person-to-person transmission risk of ANDV is essential for preparedness against future outbreaks.

Appendix Additional information about characteristics and superspreading potential of Andes virus person-to-person transmission.
