# Supplementary material for: Characteristics and Superspreading Potential of Andes Virus Person-to-Person Transmission
Source: Emerg Infect Dis. 2026 Oct;32(10):1695–8. doi: 10.3201/eid3210.260908 (PMC13632879; doi:10.3201/eid3210.260908)
Supplement: Supplementary file 1 [file 26-0908-Techapp-s1.pdf]

Article DOI: https://doi.org/10.3201/eid3210.260908

EID cannot ensure accessibility for supplementary materials supplied by authors. Readers who have difficulty accessing supplementary content should contact the authors for assistance.

Characteristics and Superspreading Potential of Andes Virus Person-to-Person Transmission

Appendix

Person-to-Person Transmission Events and Epidemiological Data in Argentina

From the literature review, we identified 5 studies that reported potential person-to-person transmission of ANDV infection in Argentina (*1*–*5*). These studies were included in the present analysis because they provided both viral lineage information and clear epidemiological links supporting person-to-person transmission based on detailed contact tracing history.

From these studies, we excluded cases with unknown contact history, either because they were used for nucleotide sequence comparison or because epidemiological links could not be established due to recall limitations. Among a total of 88 included case-clusters (34 clusters with strain Epuyén/18-19, 26 with lineage ANDV Sout, 18 with strain Epilink/96, and 10 with lineage ANDV Cent BsAs), we identified 38 case-clusters associated with secondary person-to-person transmission events, with numbers of secondary cases ranging from 1 to 10. The remaining 50 case-clusters did not generate any secondary infections, comprising 40 terminal cases (i.e., secondary cases with known primary cases but without onward transmission) and 10 sporadic cases with 0 human contact and thus also without onward transmission. Among 38 case-clusters with secondary transmissions, 76 transmission pairs (40 pairs with Epuyén/18–19, 13 with ANDV Sout, 22 with strain Epilink/96, and 1 with ANDV Cent BsAs) were obtained for estimating the serial interval distribution.

These case-clusters were grouped according to the viral lineage or strain reported in the original studies: Epuyén/18–19 strain (*3*), Epilink/96 strain (*5*), ANDV Sout lineage (*1*,*2*,*4*), and ANDV Cent BsAs lineage (*1*). The specific strain types - Epuyén/18–19 and Epilink/96, which

belong to the ANDV Sout lineage - were reported in only two of the included studies. Therefore, this grouping (rather than aggregating all records) was chosen to account for potential epidemiological differences between the Epuyén/18–19 and Epilink/96 strains and other viruses within the ANDV Sout lineage, as well as differences between the ANDV Sout lineage and the ANDV Cent BsAs lineage.

A curated dataset enabling reconstruction of historical ANDV case-clusters is available at: https://github.com/Jason66667/Transmission-characteristics-and-superspreading-potential-of-Andes-virus-person-to-person-transmission.

Statistical Inference Framework

To quantify the transmission heterogeneity, we fitted the number of secondary cases within case-clusters to a negative binomial (NB) distribution to account for overdispersion and heterogeneity in person-to-person transmission of ANDV, as suggested by previous studies (*3*). The NB distribution was parameterized by the effective reproduction number (R_t) and an overdispersion parameter (k) (*6*). The parameter R_t represents the mean number of secondary cases generated by one primary case, while k quantifies the degree of variation in individual transmissibility. A smaller value of k indicates greater transmission heterogeneity. The probability mass function of NB distribution is given by:

$$P_Z(Z = z_u; R_t, k) = \frac{\Gamma(k + z_u)}{\Gamma(k) \cdot \Gamma(z_u + 1)} \cdot \left(\frac{R_t}{R_t + k}\right)^{z_u} \cdot \left(\frac{k}{R_t + k}\right)^{k}$$

Here, z_u is the number of secondary cases caused by the primary case u, and $\Gamma(.)$ denotes the gamma function. Note that z_u can be obtained by using the documented individual-based contact tracing history for ANDV cases.

The serial interval (SI), defined as the time between symptom onset in a primary case and symptom onset in a secondary case, is a key epidemiological parameter that determines the speed of transmission (*7*). We assumed that the SI of person-to-person transmission of ANDV followed a gamma distribution, given its flexibility and the relatively long SIs observed in historical records. The probability density function of the gamma distribution is given by:

$$P_T\left(T = t_j; \mu, \sigma\right) = \frac{\left(\frac{\mu}{\sigma^2}\right)^{\left(\frac{\mu}{\sigma}\right)^2}}{\Gamma\left(\left(\frac{\mu}{\sigma}\right)^2\right)} \cdot t_j^{\left(\frac{\mu}{\sigma}\right)^2 - 1} \cdot \exp\left(^{-\frac{\mu}{\sigma^2} \cdot t_j}\right)$$

Here, exp(.) is the exponential function, t_j is the observed SI for the secondary case j, and μ and σ are the mean and standard deviation of the gamma distribution.

Both estimation of transmission heterogeneity (*R_t* and *k*) and SI distribution need contact tracing data involving "pairwise data" between infector and infectee, i.e., transmission pairs. However, such pairwise data may not always be available. When unique epidemiological links could not be established within a cluster (e.g., when a secondary case had contact with multiple primary cases) (*3*,*5*), we weighted the contribution of each primary case to the cluster size and SI by assuming equal probability of transmission from each potential infector (*8*). The likelihood functions are given by:

$$L_Z(R_t, k) = \prod_{m=1}^{M} \exp\left(\sum_{u=1}^{U} w_u \cdot \log\left(P_Z\left(z_{u,m}; R_t, k\right)\right)\right)$$

and

$$L_T(\mu, \sigma) = \prod_{j=1}^{J} \exp\left(\sum_{h=1}^{H} w_h \cdot \log\left(P_T\left(t_{h,j}; \mu, \sigma\right)\right)\right)$$

Here, log(.) is the natural logarithm function. $L_Z(R_t, k)$ is the likelihood for the total *M* case-clusters, with $z_{u,m}$ denoting the number of secondary cases caused by primary case u in the m-th case-cluster that contains a total of U primary cases ($U \geq 1$), and w_u denoting the probability that the u-th primary case had the infectious contacts ($w_u = \frac{1}{u}$) in the m-th case-cluster. Likewise, $L_T(\mu, \sigma)$ is the likelihood of observing SIs for a total of J secondary cases, with t_h denoting the SI for the j-th secondary case originated from the h-th primary case out of a total of H primary cases ($H \geq 1$) that had contacts with the j-th secondary case, and w_h denoting the probability that the h-th primary case had the true epidemiological link ($w_h = \frac{1}{H}$) with the j-th secondary case. There were eight out of the total 88 case-clusters (9%) involved multiple primary cases (i.e., $U > 1$ or $H > 1$).

We used a Bayesian hierarchical model (*9*) to obtain pooled estimates of R_t and μ while accounting for potential random effects arising from inherent epidemiological differences between ANDV lineages or strains. Specifically, we allowed each lineage or strain g to have a deviation from the overall pooled estimate (d_g). We assumed that these deviations followed a normal distribution with a mean of 0 and a standard deviation of s. Thus, the lineage- or strain-specific estimate was given by the sum of the pooled estimate and the corresponding deviation d_g.

Estimation Procedure

We estimated the models' parameters by using the Markov chain Monte Carlo (MCMC) method using the "*rstan*" package (*10*). We used a weakly informative prior Cauchy(0, 5) for s and non-informative flat priors for transmission heterogeneity parameters R and k, and SI distribution parameters μ and σ. Marginal posterior distributions were obtained from 4 MCMC chains with 50000 iterations for each chain, and the first 2000 samples were discarded as warm-up iterations. The convergence of each MCMC chain was checked by using the trace plot and Gelman-Rubin-Brooks convergence diagnostic. The median as well as the 95% credible interval (CrI) of posterior samples were calculated for each model parameter.

Outbreak Trajectory Simulations and Analysis of Transmission Heterogeneity

As of July 2, 2026, a total of 13 case-patients have been identified in the cruise ship outbreak caused by person-to-person transmission of ANDV, according to the World Health Organization (WHO) (*11*). To provide insights into the transmission potential of the cruise ship outbreak, we employed a stochastic branching process model with negative binomial distributed offspring case numbers that were parametrized by the initial reproduction number and the pooled k estimates (considering potential transmission heterogeneity) to simulate outbreak trajectories.

Before the release of the initial WHO outbreak notification and the associated public health response on 2 May, eight cases occurred within approximately one mean serial interval (18–25 days, consistent with our pooled SI estimates [Figure 1, panel B]) following the putative seed case's symptom onset (*12*). Under these conditions, conventional methods cannot reliably

estimate the initial reproduction number, as they rely on strong assumptions regarding the epidemic curve and intervention timing (*13*,*14*).

We assumed the initial reproduction number for the current ANDV outbreak lies within the range 0.4–2.5, encompassing historical point estimates of person-to-person transmission (*3*). In each simulation, the initial reproduction number was randomly drawn from a loguniform distribution to ensure equal sampling probability on either side of the epidemic threshold ($R_t = 1$) (Appendix Figure 1). The corresponding probability density function is:

$$f\left(R_{t\_initial}; a, b\right) = \frac{1}{R_{t\_initial} \cdot \log(\frac{b}{a})}$$

Here, $R_{t\_initial}$ denotes the initial reproduction number; a and b denote the lower and upper boundaries of the loguniform distribution, which are equal to 0.4 and 2.5, respectively.

Consistent with the current situation, a single seed case (with symptom onset on April 3, 2026, as per (*11*)) was assumed to initiate the outbreak. Symptom onset dates for subsequent cases were sampled from the pooled SI distribution. According to the initial WHO notification for the outbreak on May 2, 2026 (*12*), public health and control measures, including contact tracing, case isolation, clinical care, and medical evacuation of symptomatic passengers, have been implemented on board. We assumed that effective public health control measures were implemented immediately after May 2, 2026, after which the transmission potential was assumed to decrease, with the effective reproduction number drawn from a uniform distribution, i.e., Uniform(0, 0.5), for onward transmission. We performed 1,000 stochastic simulations, each running for up to 10 weeks after the seed case symptom onset.

To further analyze the transmission heterogeneity, we calculated the expected proportion of cases responsible for 80% of all transmission events (*6*). We also estimated the probability that a transmission chain would be sustained after a given number of generations, using both the assumed initial reproduction number for the cruise ship outbreak and the pooled R_t from historical clusters (*15*).

Sensitivity Analysis

We conducted several sensitivity analyses to test the robustness of our findings. First, we excluded sporadic cases that had no documented contact with any infected cases from the included case-clusters. This provided estimates that are more generalizable to the context of person-to-person transmission in a close-contact setting, consistent with the current cruise ship outbreak. Second, for the simulated cruise ship outbreak, we examined alternative assumptions regarding public health control measures by assuming 1) no secondary cases are produced by cases with symptom onset after May 2, 2026; 2) public health control measures are implemented two weeks later than the main scenario (i.e., implemented on May 16, 2026); and 3) no public health control measures are implemented.

All analyses were performed using R statistical software (version 4.4.2).

References

1. Martinez VP, Bellomo C, San Juan J, Pinna D, Forlenza R, Elder M, et al. Person-to-person transmission of Andes virus. Emerg Infect Dis. 2005;11:1848–53. PubMed https://doi.org/10.3201/eid1112.050501

2. Lázaro ME, Cantoni GE, Calanni LM, Resa AJ, Herrero ER, Iacono MA, et al. Clusters of hantavirus infection, southern Argentina. Emerg Infect Dis. 2007;13:104–10. PubMed https://doi.org/10.3201/eid1301.060404

3. Martínez VP, Di Paola N, Alonso DO, Pérez-Sautu U, Bellomo CM, Iglesias AA, et al. "Super-spreaders" and person-to-person transmission of Andes virus in Argentina. N Engl J Med. 2020;383:2230–41. PubMed https://doi.org/10.1056/NEJMoa2009040

4. Alonso DO, Pérez-Sautu U, Bellomo CM, Prieto K, Iglesias A, Coelho R, et al. Person-to-person transmission of Andes virus in hantavirus pulmonary syndrome, Argentina, 2014. Emerg Infect Dis. 2020;26:756–9. PubMed https://doi.org/10.3201/eid2604.190799

5. Wells RM, Sosa Estani S, Yadon ZE, Enria D, Padula P, Pini N, et al. Hantavirus Pulmonary Syndrome Study Group for Patagonia. An unusual hantavirus outbreak in southern Argentina: person-to-person transmission? Emerg Infect Dis. 1997;3:171–4. PubMed https://doi.org/10.3201/eid0302.970210

6. Lloyd-Smith JO, Schreiber SJ, Kopp PE, Getz WM. Superspreading and the effect of individual variation on disease emergence. Nature. 2005;438:355–9. PubMed https://doi.org/10.1038/nature04153

7. Fine PEM. The interval between successive cases of an infectious disease. Am J Epidemiol. 2003;158:1039–47. PubMed https://doi.org/10.1093/aje/kwg251

8. Perez-Saez J, Bugeme PM, O'Driscoll M, Bugale PK, Mukika TF, Bugwaja L, et al. The incubation periods of mpox virus clade Ib. Ann Intern Med. 2026;179:207–15. PubMed https://doi.org/10.7326/ANNALS-25-01016

9. Miura F, Backer JA, van Rijckevorsel G, Bavalia R, Raven S, Petrignani M, et al.; Dutch Mpox Response Team. Time scales of human mpox transmission in the Netherlands. J Infect Dis. 2024;229:800–4. PubMed https://doi.org/10.1093/infdis/jiad091

10. Carpenter B, Gelman A, Hoffman MD, Lee D, Goodrich B, Betancourt M, et al. Stan: a probabilistic programming language. J Stat Softw. 2017;76:1–32. PubMed https://doi.org/10.18637/jss.v076.i01

11. World Health Organization. Hantavirus outbreak linked to cruise ship travel, multi-locations. 2026 Jul 2 [cited 2026 Jul 8]. https://www.who.int/emergencies/disease-outbreak-news/item/2026-DON611

12. World Health Organization. Hantavirus cluster linked to cruise ship travel, multi-country. 2026 May 4 [cited 2026 Jul 8]. https://www.who.int/emergencies/disease-outbreak-news/item/2026-DON599

13. Obadia T, Haneef R, Boëlle P-Y. The R0 package: a toolbox to estimate reproduction numbers for epidemic outbreaks. BMC Med Inform Decis Mak. 2012;12:147. PubMed https://doi.org/10.1186/1472-6947-12-147

14. Blumberg S, Lloyd-Smith JO. Inference of $R_{(0)}$ and transmission heterogeneity from the size distribution of stuttering chains. PLOS Comput Biol. 2013;9:e1002993. PubMed https://doi.org/10.1371/journal.pcbi.1002993

15. Nishiura H, Miyamatsu Y, Chowell G, Saitoh M. Assessing the risk of observing multiple generations of Middle East respiratory syndrome (MERS) cases given an imported case. Euro Surveill. 2015;20:21181. PubMed https://doi.org/10.2807/1560-7917.ES2015.20.27.21181

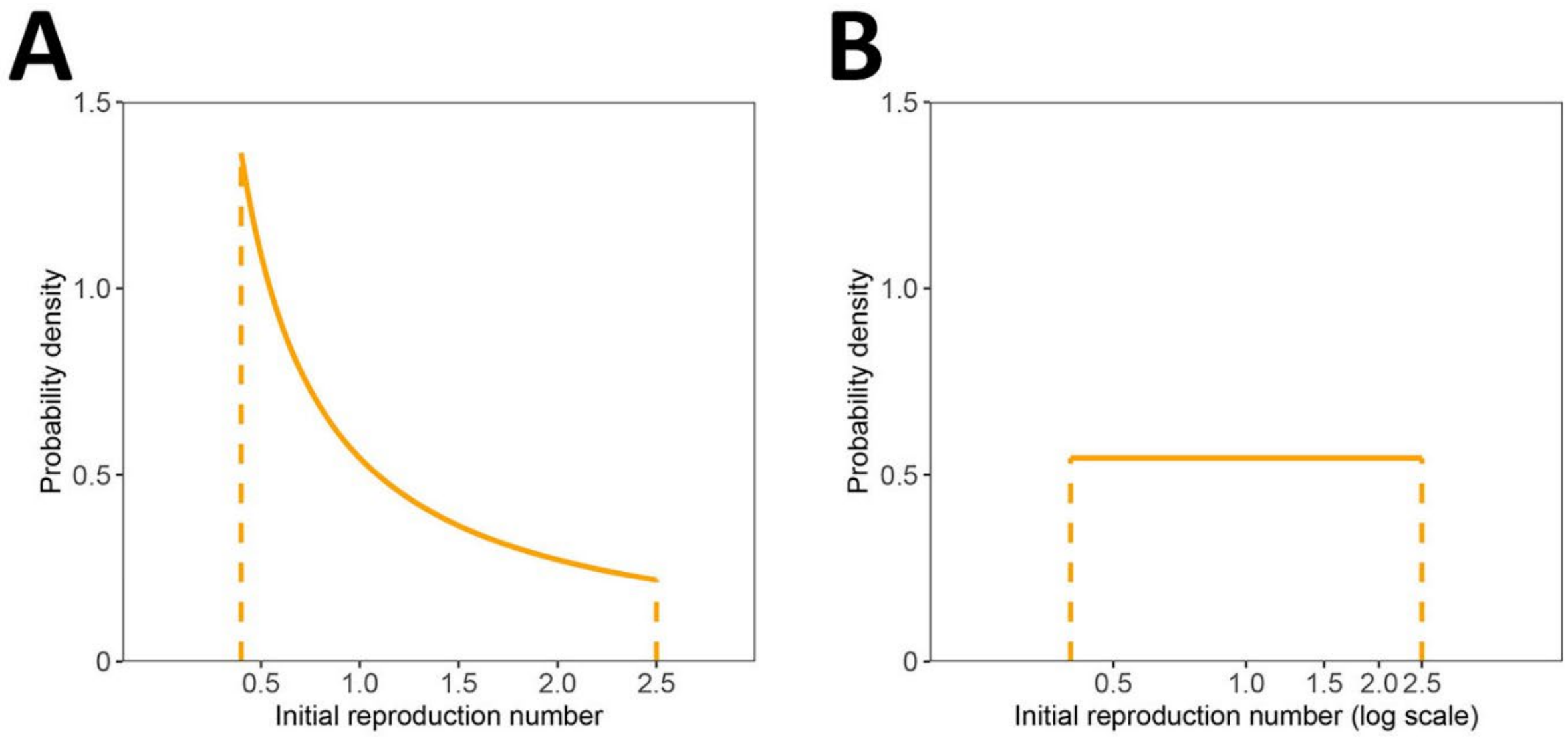

Appendix Figure 1. Probability density function of loguniform distribution loguniform(0.4, 2.5). A) The initial reproduction number of the current ANDV outbreak was assumed to lie within the range 0.4–2.5 and was randomly sampled from loguniform(0.4, 2.5) in each outbreak simulation. B) The same density plotted with the initial reproduction number shown on a log scale.

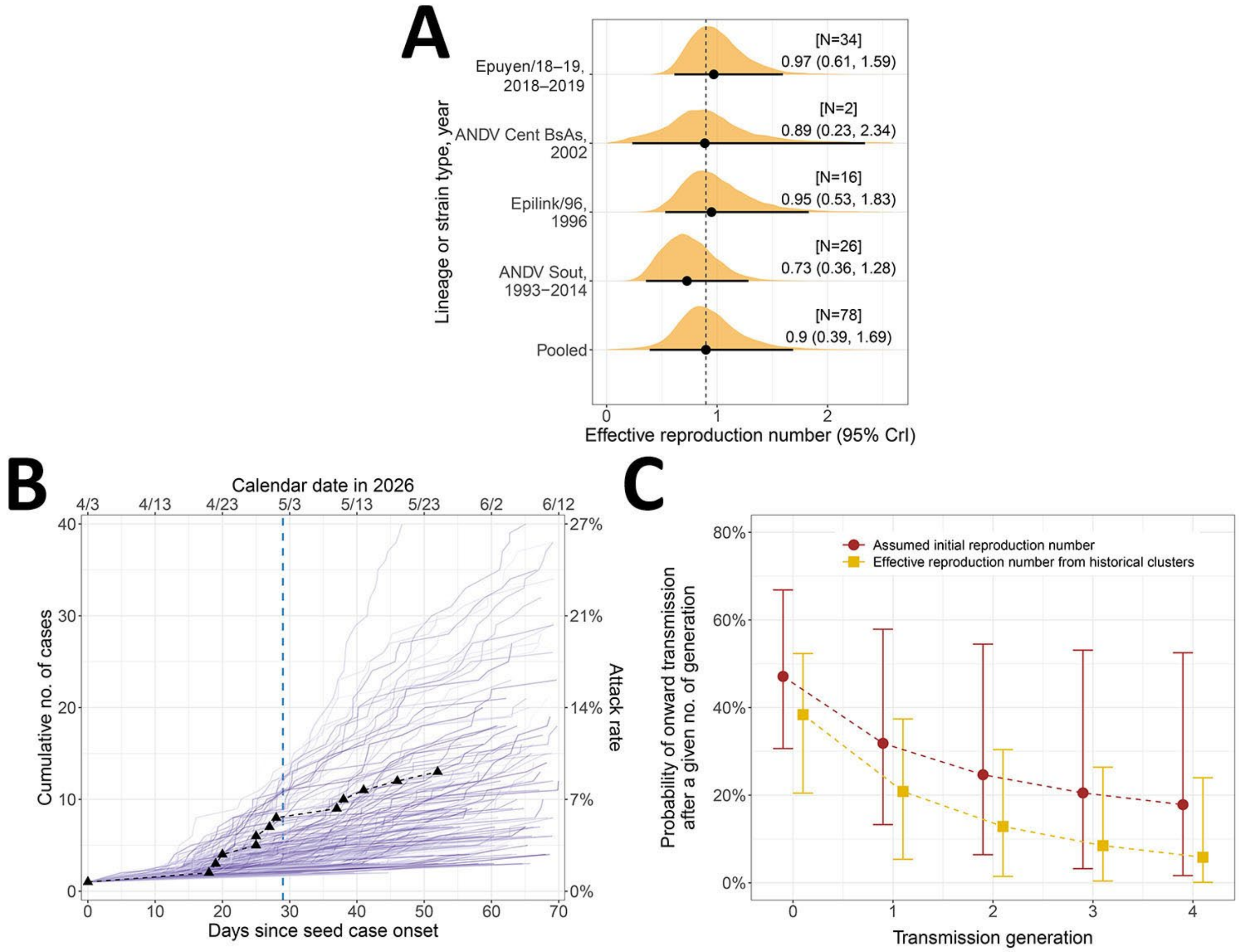

Appendix Figure 2. Sensitivity analysis of R_t and *k* estimates from historical ANDV clusters by excluding sporadic cases. A) The pooled R_t estimated from historical interhuman transmission events, incorporating random effects from different ANDV lineages or strains reported in outbreaks that occurred in previous years. B) Projected outbreak trajectories from 1000 branching process simulations within 10 weeks after symptom onset of the seed case (April 3, 2026), using a plausible range of the initial reproduction number of the cruise ship cluster (0.4–2.5) and pooled dispersion parameter (*k*) estimates from historical data. Symptom onset dates for secondary cases were randomly sampled from the pooled SI distribution. The triangular dots indicate the cumulative number of cases observed in the cruise ship outbreak as of May 27, 2026. The blue dashed vertical line marks the initial WHO notification date of the outbreak (May 2, 2026), after which effective control measures were assumed to have been implemented (i.e., the reproduction number was assumed to decrease). The right y-axis shows the corresponding attack rate based on a ship population of 147. C) Probability of onward transmission after a given number of generations of transmission, estimated using the assumed plausible initial reproduction number for the cruise ship cluster, the pooled R_t, and the *k* estimates from historical clusters. In panels A and C, dots represent mean estimates and whiskers indicate 95% CrI.

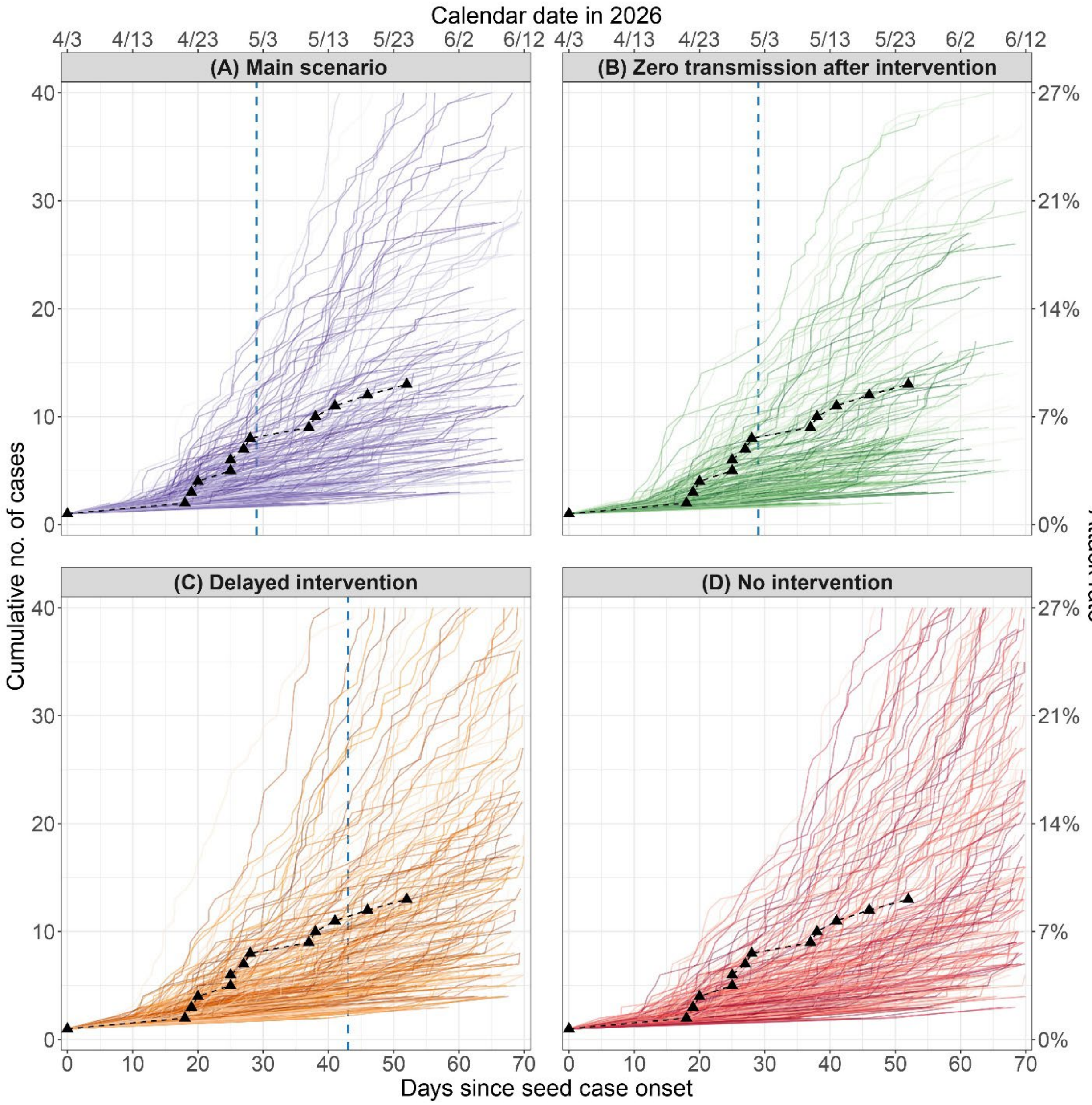

Appendix Figure 3. Sensitivity analysis of the cruise ship outbreak simulation using alternative assumptions about public health control measures, compared with the main analysis. A) Main analysis scenario with a reduction in the reproduction number for cases with symptom onset after May 2, 2026. B) No transmission occurred after May 2, 2026. C) Public health control measures are implemented with a 2-week delay relative to the main scenario (May 16, 2026). D) No public health control measures are implemented. In all panels, projected outbreak trajectories were generated from 1000 branching process simulations within 10 weeks after symptom onset of the seed case (April 3, 2026), using the assumed plausible initial reproduction number for the cruise ship cluster (0.4–2.5) and pooled dispersion parameter (*k*) estimates from historical data. Symptom onset dates for secondary cases were randomly sampled from the pooled SI distribution. The triangular dots indicate the cumulative number of cases observed in

the cruise ship outbreak as of May 27, 2026. The right y-axis shows the corresponding attack rate based on a ship population of 147.
